# Intermittent fasting—a double edged sword for atherosclerosis

**DOI:** 10.1093/lifemeta/loae015

**Published:** 2024-04-25

**Authors:** Jacques Togo, Hoon-Ki Sung

**Affiliations:** Translational Medicine Program, The Hospital for Sick Children, Toronto, Ontario M5G 0A4, Canada; Translational Medicine Program, The Hospital for Sick Children, Toronto, Ontario M5G 0A4, Canada; Department of Laboratory Medicine and Pathobiology, University of Toronto, Toronto, Ontario M5S 1A8, Canada

**The impact of alternate-day fasting (ADF) on atherosclerosis remains poorly understood, particularly in high-risk populations. In a recent paper published in *Life Metabolism*, Deng *et al.* revealed that ADF exacerbated both early and advanced atherosclerotic lesion development in *Apoe***^***−/−***^ **mice through suppression of hepatic activating transcription factor 3 (ATF3) and consequent dysregulation of cholesterol metabolism.**

Atherosclerosis, a condition characterized by the accumulation of fatty deposits in the inner lining of arteries, leading to narrowing over time, is a primary pathology underlying cardiovascular diseases (CVDs) [1]. Despite its significant impact, current therapeutic approaches remain unsatisfactory due to the complex nature and multifactorial etiology of the disease. Intermittent fasting (IF) has emerged as a potential dietary strategy, involving cycles of fasting and unrestricted eating. Alternate-day fasting (ADF), a popular IF regimen, has gained attention for its various metabolic benefits, including weight loss and improving glucose and insulin sensitivity. Moreover, ADF is often considered a more manageable and potentially superior alternative to other dietary interventions, such as calorie restriction [2]. Nevertheless, the impact of IF on atherosclerosis remains controversial and largely unexplored [3, 4].

One of the most significant advancements in understanding the factors influencing the development of atherosclerosis has been the establishment of mouse models for studying this condition. Among the various models available, the apolipoprotein E-deficient (*Apoe*^*−/−*^) mouse is one of the most widely utilized. Apolipoprotein E (ApoE) is a pivotal player in lipid metabolism by facilitating the binding of lipoproteins or lipid complexes in the plasma to cell-surface receptors. This process enables the internalization of ApoE-containing lipoprotein particles, thereby aiding in the redistribution of lipids in the body. Predominantly synthesized by the liver and macrophages in peripheral tissues, ApoE is vital for efficient clearance of chylomicrons and remnants of very low-density lipoproteins (VLDL) by the liver and adipose tissue [5].

*ApoE* knockout mice have served as a cornerstone in atherosclerosis research due to their propensity to develop severe hypercholesterolemia and atherosclerosis when subjected to either a high-fat diet or chow diet, making them a useful tool for studying the mechanisms underlying atherosclerotic lesion formation and evaluating the efficacy of potential therapeutic interventions.

**Figure 1:**
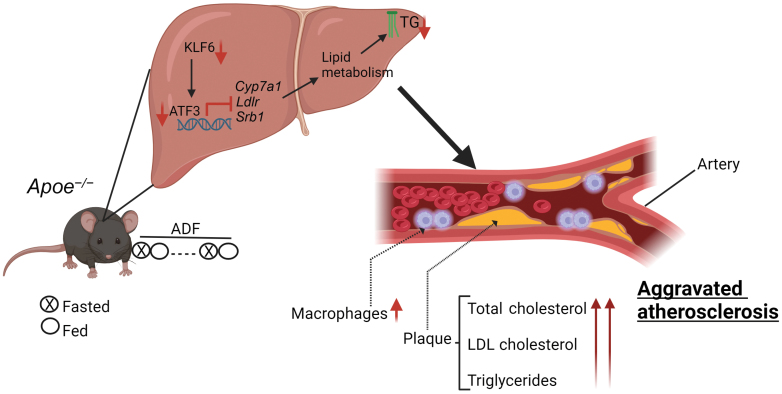
ADF aggravates atherosclerosis in *ApoE* deficient mice subjected to the Western diet. ADF suppresses cholesterol metabolism-related genes through the inhibition of ATF3. Increased infiltration of macrophages indicates increased lesion formation. TG, triglyceride; ADF, alternate-day fasting. Created with Biorender.com.

In this study by Deng *et al*., 11-week-old male *Apoe*^*−/−*^ mice were subjected to a Western diet (WD) for 8 weeks [6], with or without concurrent administration of atorvastatin (ATOR), a widely used statin medication known for its cholesterol-lowering effects and its ability to mitigate cardiovascular risks in patients with atherosclerosis [7]. Surprisingly, the findings indicated that ADF exacerbated both early and advanced atherosclerotic lesion formation. Specifically, compared to control mice, ADF led to a significant augmentation of lesion area and size. In a more advanced atherosclerotic model following 16 weeks of WD, ADF induced larger lesions in both aortas and aortic roots, regardless of ATOR treatment. Furthermore, ADF increased the accumulation of macrophage and vascular muscle cells within atherosclerotic lesions, thereby exacerbating inflammatory profiles and atherosclerotic progression in *Apoe*^*−/−*^ mice (Fig. 1).

At this point in the story, the question of whether ADF-mediated exacerbation of atherosclerosis occurs independently of the overall metabolic benefits remains unknown, given previous reports suggesting that ADF elicits metabolic benefits. Intriguingly, the current study also found that ADF reduces body weight gain and adiposity, improves glucose homeostasis, and ameliorates diet-induced fatty liver, as evidenced by decreased hepatic triglycerides compared with the control mice. In line with findings from numerous prior studies, the study further noted a reduction in adipocyte size, a hallmark of healthy adipose remodeling and indicative of efficient lipid storage within adipocytes, potentially mitigating ectopic lipid deposition, such as in the liver.

Adipose tissue plays a crucial role in maintaining whole-body energy homeostasis by orchestrating lipid mobilization and distribution throughout the body. Moreover, under certain conditions, such as cold exposure or IF, brown and beige adipose tissues engage in lipid burning, generating heat to regulate body temperature. Notably, Deng *et al*. utilized indirect calorimetry, showing that ADF increased energy expenditure and lowered respiratory exchange ratio in *Apoe*^*−/−*^ mice, indicative of enhanced lipid utilization as the main fuel source. Moreover, genes associated with thermogenesis and browning of white adipose tissue, including cell death-inducing DNA fragmentation factor-like effector A (*Cidea*), peroxisome proliferator-activated receptor-γ coactivator-1α (*Pgc1α*), and uncoupling protein 1 (*Ucp1*), were upregulated in ADF group. These findings suggest that the aggravation of atherosclerosis in *Apoe*^*−/−*^ mice under ADF was not due to WD-induced obesity, highlighting the complex interplay between ADF and atherosclerosis in this model.

Having established this foundation, Deng *et al*. then proceeded to explore the complexity of the effects of ADF on lipid metabolism and atherosclerosis in male and female *Apoe*^*−/−*^ mice, as sex differences have been observed in the development and progression of this condition [8]. Despite the protective effects against WD-induced obesity mentioned earlier, ADF led to increased circulating levels of total cholesterol (TC), total triglycerides (TGs), low-density lipoprotein cholesterol (LDL-C), high-density lipoprotein cholesterol (HDL-C), and VLDL in both sexes. As the liver plays a crucial role in cholesterol metabolism, the authors performed transcriptome analysis of this tissue and revealed significant changes in gene expression related to cholesterol metabolism. While the liver is primarily responsible for synthesizing and processing endogenous cholesterol, intestinal enterocytes also play a role in cholesterol absorption. However, ADF did not influence intestinal cholesterol absorption, suggesting that its effects on atherosclerosis in *Apoe*^*−/−*^ mice are mediated through altered liver cholesterol metabolism.

One of the most recently recognized factors regulating cholesterol metabolism is the activating transcription factor 3 (ATF3). ATF3 influences lipoprotein metabolism by regulating key genes involved in cholesterol biogenesis and metabolism, including cholesterol 7a-hydroxylase (*Cyp7a1*), low-density lipoprotein receptor (*Ldlr*), and scavenger receptor group B type 1 (*Srb1*) [8]. ATF3 serves as a master regulator in metabolic homeostasis and is known to upregulate genes involved in lipolysis and browning in adipocytes. Xu *et al*. reported that ATF3 enhances hepatic HDL uptake and protects against hepatic cholesterol accumulation [9]. In their investigation of hepatic cholesterol metabolism, Deng *et al*. found that ADF suppressed the expression of ATF3 in *Apoe*^*−/−*^ mice. Furthermore, the decrease in mRNA levels of ATF3 network components, such as *Cyp7a1*, *Ldlr*, and *Srb1*, in ADF-treated *Apoe*^*−/−*^ mice suggested that hepatic ATF3 expression is pivotal to altered cholesterol metabolism observed in these mice (Fig. 1).

To elucidate the specific role of hepatic ATF3 in ADF-mediated atherosclerosis development, the authors utilized an adeno-associated virus (AAV) system (AAV8-TBG-ATF3) to restore ATF3 expression specifically in the liver of *Apoe*^*−/−*^ mice under ADF. Interestingly, ADF led to reduced atherosclerotic plaque formation, and decreased hepatic cholesterol content and serum levels of TC and LDL-C compared to the control. Moreover, *Atf3* overexpression upregulated key cholesterol metabolism genes, including *Cyp7a1* and *Ldlr*. CYP7A1 is an enzyme that controls the pace of converting cholesterol into bile acids that lead to reductions in TC and LDL-C levels. As to LDLR, it is vital in modulating plasma LDL-C levels by facilitating its uptake into hepatocytes, thereby enhancing the clearance of circulating LDL-C and potentially reducing atherosclerosis progression [10]. These results, therefore, indicate that ATF3 is a key player in mitigating ADF-induced atherosclerosis via modulation of cholesterol metabolism.

The authors then delved into the mechanisms of ATF3 regulation by the known integrated stress response (ISR) pathway, which is suppressed in their study during ADF intervention. ISR is a signaling pathway that is activated in response to diverse stressors. In addition, a recent study suggested that Krüppel-like factor 6 (KLF6), a transcription factor known to respond to oxidative stress in pathological conditions, may regulate ATF3 expression in the context of hepatic ISR, by directly binding to and activating *Atf3* promoter, potentially linking these two pathways in the regulation of cellular stress responses and metabolic homeostasis [11]. However, the specific regulatory mechanisms of KLF6 on ATF3 in hepatic ISR remain unclear. Finally, Deng *et al*. found a significant positive correlation between *Atf3* and *Klf6* expression in hepatic ISR, indicated by RNA sequencing (RNA-seq) data. Furthermore, experiments using mouse hepatocytes treated with tunicamycin, an ISR stressor, demonstrated that both KLF6 and ATF3 expressions were induced, and this induction was reversed by ISRIB, an ISR inhibitor. Knockdown of *Atf4*, a key ISR effector, attenuated the induction of KLF6 and ATF3, suggesting that ADF inhibits hepatic ISR and reduces KLF6 and ATF3 expression through ATF4. The authors further validated their findings through direct KLF6 gain-of-function and loss-of-function studies in hepatocytes and confirmed its role in ATF3 regulation. These findings highlight the role of KLF6 in regulating ATF3 expression during hepatic ISR, providing insights into the regulatory network that controls ADF-mediated lipid metabolism dysregulation leading to atherosclerosis development.

The study by Deng *et al*. provides compelling evidence of a previously unappreciated impact of ADF on atherosclerosis development. The study demonstrates that ADF exacerbates early and advanced atherosclerotic lesion formation in mice lacking ApoE, potentially due to disturbances in cholesterol profiles. Mechanistically, ADF inhibits the hepatic ISR signaling pathway, leading to reduced expression of KLF6 and consequent inhibition of ATF3 expression. The suppressed ATF3 expression in the liver is implicated in the exacerbated effects of ADF on atherosclerosis in these mice. However, the research has several limitations. Although changes in cholesterol profiles were observed after ADF, the researchers did not measure the rates of cholesterol synthesis in the liver or intestinal cholesterol absorption to validate the source of increased circulating cholesterol levels. In addition, the regulatory effects of KLF6 on ATF3 should be further confirmed in ISR pathways, as suggested. While the study was conducted in animal models, which may not directly translate to humans, it serves as a cautionary reminder that dietary interventions like ADF should be approached with caution, especially for individuals at high risk of atherosclerosis, such as those carrying the ApoE-ε4 allele.
